# Immobilization of enzyme and antibody on ALD-HfO_2_-EIS structure by NH_3 _plasma treatment

**DOI:** 10.1186/1556-276X-7-179

**Published:** 2012-03-08

**Authors:** I-Shun Wang, Yi-Ting Lin, Chi-Hsien Huang, Tseng-Fu Lu, Cheng-En Lue, Polung Yang, Dorota G Pijanswska, Chia-Ming Yang, Jer-Chyi Wang, Jau-Song Yu, Yu-Sun Chang, Chien Chou, Chao-Sung Lai

**Affiliations:** 1 Institute of Electro-Optical Engineering, Chang Gung University, 259 Wen-Hwa 1st Road, Kwei-Shan, Tao-Yuan, 333, Taiwan; 2 Department of Electronic Engineering, Chang Gung University, 259 Wen-Hwa 1st Road, Kwei-Shan, Tao-Yuan, 333, Taiwan; 3 Molecular Medicine Research Center, Chang Gung University, 259 Wen-Hwa 1st Road, Kwei-Shan, Tao-Yuan, 333, Taiwan; 4 Nalecz Institute of Biocybernetics and Biomedical Engineering, Polish Academy of Sciences, Warsaw, 02-668, Poland; 5 Biosensor Group, Biomedical Engineering Center, Chang Gung University, 259 Wen-Hwa 1st Road, Kwei-Shan, Tao-Yuan, 333, Taiwan

**Keywords:** remote plasma, silanization procedure, surface functionalization

## Abstract

Thin hafnium oxide layers deposited by an atomic layer deposition system were investigated as the sensing membrane of the electrolyte-insulator-semiconductor structure. Moreover, a post-remote NH_3 _plasma treatment was proposed to replace the complicated silanization procedure for enzyme immobilization. Compared to conventional methods using chemical procedures, remote NH_3 _plasma treatment reduces the processing steps and time. The results exhibited that urea and antigen can be successfully detected, which indicated that the immobilization process is correct.

## Introduction

The variation of human body fluid in tiny concentrations can be critical for clinical diagnosis. Therefore, the detection of chemical and biological species through microelectronic sensor devices has attracted great attention over the past decade. Ion-sensitive field-effect transistors [ISFETs] are one of the silicon-based potential metric sensors with the advantages of compatibility and integration with advanced complementary metal-oxide-semiconductor processes and cost reduction. Until now, plenty of high-*k *materials have been applied to the sensing membranes of ISFETs, including SiO_2_, Si_3_N_4 _[1,2], Ta_2_O_5 _[3-5], Al_2_O_3 _[6], TiO_2 _[7,8], HfO_2 _[9,10], SnO_2 _[11], etc. Among numerous proposed high-*k *materials, hafnium oxide [HfO_2_], characterized by high pH sensitivity, low drift, low hysteresis, and low body effect, is a promising pH-sensing material [9,12]. In recent years, there are more and more developments on ISFETs such as the chemical field-effect transistor, enzymatic field-effect transistor [EnFET], Bio-FET [13], DNAFET, etc. For the purpose of monitoring the small changes in body fluid during the early stages, an accurate and stable sensor is needed.

As mentioned above, EnFET is one of the sensors for many biomarkers. The earliest report of EnFET was proposed by Caras and Janata in 1980 [14]. Subsequently, many biomarkers have been detected by EnFET, such as penicillin [14], urea [15], glucose [16], creatinine [17], etc. To fabricate the EnFET, a specific enzyme is immobilized on the surface of the sensing membrane of an ISFET. Moreover, to immobilize biomolecules (such as enzymes, antibodies, and probe-DNAs) [18] for monitoring the biomarkers (antigens and target-DNAs), many approaches have been developed, including physical adsorption [19,20], covalent bonding [21], entrapment [22], etc. However, the silanization procedure for producing reactive groups (NH_2_) on the material is time-consuming and complicated.

In this paper, the pH sensing properties of HfO_2 _sensing layers with various thicknesses were fabricated by an atomic layer deposition [ALD] system and investigated by an electrolyte-insulator-semiconductor [EIS] structure. The EIS structure is a capacitive sensor in which the changes in surface potential between the electrolyte and the sensing insulator could be measured according to the shift of capacitance-voltage [C-V] curves. Compared to the complex processing of the ISFET, EIS is one of the simplest platforms as an ISFET replacement for the preliminary investigation of the properties of new sensing materials. For the purpose of saving the process time of the bioreactor immobilization, HfO_2 _sensing membranes with post-ammonia [NH_3_] plasma treatment were used to replace the chemical procedures.

## Experimental process

The standard buffer solutions from pH 2 to pH 12 for the pH detection were purchased from Merck (Taipei, Taiwan). For the experiment about urea and antigen, all materials were bought from Sigma (St. Louis, MO, USA), including urease, (3-aminopropyl)triethoxysilane, glutaraldehyde [GA], urea, ethanolamine, and bovine serum albumin [BSA]. Anti-BSA is provided from the Biomedical Engineering Center of Chang Gung University. Urea and urease solutions were diluted with a phosphate buffer solution, which has been adjusted to pH 6 and pH 7.4 as a background solution for urea and BSA detection, respectively [15,17].

The EIS structures with an ALD-HfO_2 _sensing membrane were used in this study (hereafter, the sample is called ALD-HfO_2_-EIS). After standard RCA clean, thin HfO_2 _layers with different thicknesses were deposited on p-type Si wafers by an ALD system at 200°C using tetrakis(ethylmethylamino)hafnium as the precursor. H_2_O vapor served as the oxygen source, and Ar gas was supplied as the purge and carrier gas. The thicknesses of ALD-HfO_2 _films are 3.5, 5, 7.5, and 10 nm. The ALD system was initially pumped down to 1 × 10^7 ^Torr, and the working pressure was maintained at 5 × 10^-1 ^Torr with purified Ar flow of 200 sccm. Next, a 300-nm-thick aluminum (Al) film as the back-side contact was evaporated on the wafer after removing the native oxide. Hereafter, to define the sensing area, a negative photoresist SU8-2005 (MicroChem Corporation, Newton, MA, USA) was used in a standard photolithography process. Finally, the EIS structures were assembled on printed circuit boards with a silver paste (TED PELLA, Inc., Redding, CA, USA) and then encapsulated with epoxy.

**Figure 1:**
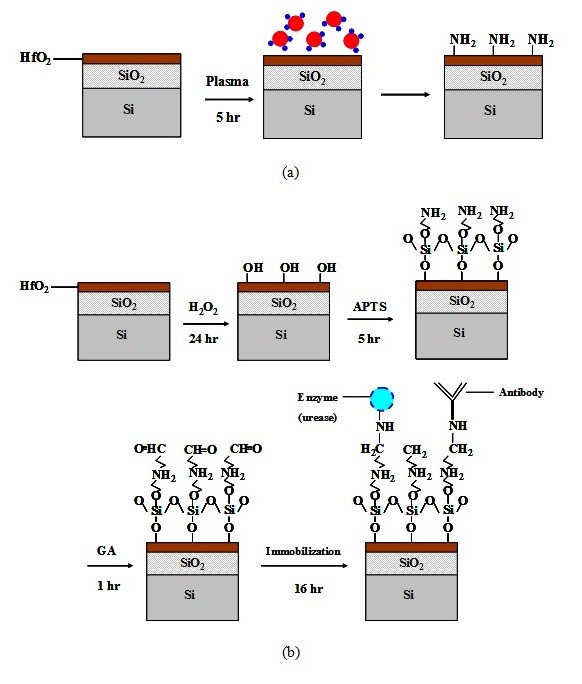
**Schematics of NH_2 _molecular and covalent bonding process flow of ALD-HfO_2_-EIS**. (**a**) NH_2 _molecular formed after plasma treatment. (**b**) Covalent bonding process flow of ALD-HfO_2_-EIS based on sensing membrane.

In order to avoid the instability from the leakage current, a 50-nm-thick buffered SiO_2 _layer was thermally grown by dry oxidation before the deposition of HfO_2 _layers. The remote NH_3 _plasma was also performed in the ALD system without breaking vacuum. The treatment was produced in Ar (25 sccm) and NH_3 _(100 sccm) ambient at 200 W for 6 min as shown in Figure 1a.

In order to compare with the samples with plasma treatment, the ALD-HfO_2_-EIS structures with conventional covalent bonding were used on the HfO_2 _layer without NH_3 _plasma treatment as the control samples as shown in Figure 1b[23]. On one hand, for urea detection, the urease powder was mixed with the phosphate buffer in a concentration of 1.5 μg/mL, and the urease was dripped on the open window of EIS before storing the sample at 4°C (in the fridge) overnight. On the other hand, for BSA detection, anti-BSA was immobilized after the sample was immersed in GA. Afterwards, ethanolamine was dripped for blocking. After rinsing the non-immobilized biomolecular by phosphate buffer, the EISs were ready for measurement.

## Results and discussion

**Figure 2:**
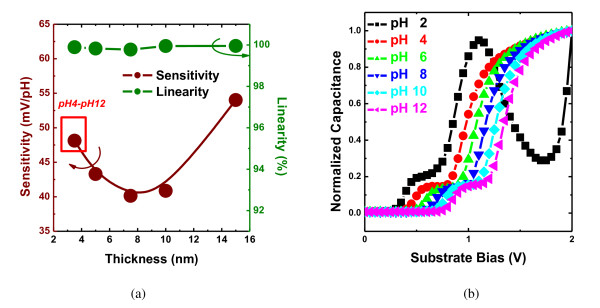
**pH Sensitivity and linearity characteristics and normalized C-V curves**. (**a**) pH sensitivity and linearity characteristics of the ALD-HfO_2_-EIS devices with various HfO_2 _thicknesses. (**b**) Normalized C-V curves for the ALD-HfO_2 _and 3.5-nm-thick ALD-HfO_2_-EIS structure measured at pH 2 to pH 12.

At first, the C-V curves of EIS structures were measured in various standard pH buffer solutions ranging from pH 2 to pH 12. The real pH value was determined using a commercial pH electrode (S120C, Sensorex, Garden Grove, CA, USA) and a pH meter (HTC-201U, HOTEC, Newton, MA, USA) before measurements. The pH sensitivity was calculated from the slope of output voltage, which is obtained at the 0.6 Cmax of the normalized C-V curves. The dependences of the calculated pH sensitivity and linearity of the ALD-HfO_2_-EIS structures with different thicknesses of HfO_2 _layers are exhibited in Figure 2a. For the thickness lower than 10 nm, the pH sensitivity is around 40 to 45 mV/pH, and for the 3.5-nm-thick ALD-HfO_2_-EIS structure, the available pH range is only from pH 4 to pH 12. Figure 2b shows the normalized C-V curves of 3.5-nm-thick ALD-HfO_2_-EIS structures, which were measured at pH 2 to pH 12. In this case, the C-V curve measured at pH 2 represents an unstable response in the accumulation region. It could be the result of the leakage current due to its flimsy thickness. The pH sensitivity is high enough (54 mV/pH) and stable when the thickness of HfO_2 _layers is higher than 15 nm. As compared to our previous study, the drift coefficient of the ALD-HfO_2_-EIS is stable and quite low (< 0.2 mV/h) when the thickness of the ALD-HfO_2 _film decreases. However, for the sputtered HfO_2_-EIS, the drift coefficient increases when the thickness of the sputtered HfO_2 _film decreases [24]. It could be that the thin HfO_2 _film prepared by ALD was much denser than that deposited by sputtering [25].

**Figure 3:**
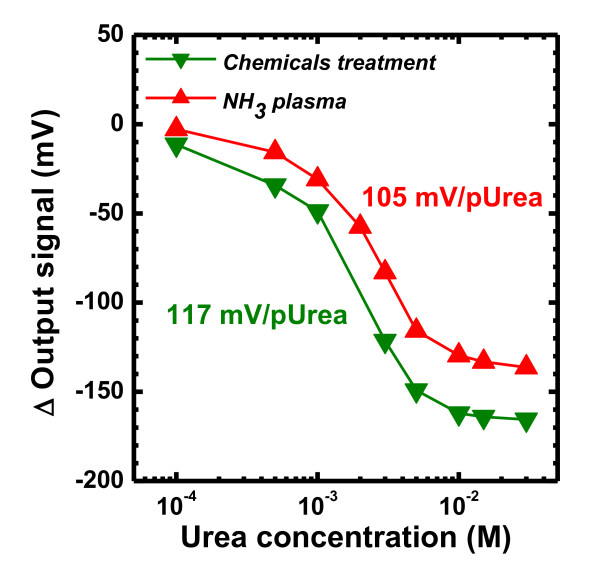
**The urea detection of ALD-HfO_2_-EIS structure with chemical silanization and NH_3 _plasma treatment**.

Considering the application on biomedical sensors, the stacked structure of 15-nm-thick HfO_2_/50-nm-thick SiO_2_/Si EIS was used. After the urease was immobilized on the surface of HfO_2 _layers with NH_3 _plasma post-treatment or the conventional silanization method, the HfO_2_-EIS structures were immersed into the PB solutions with different concentrations of urea. As shown in Figure 3, the output voltage of the HfO_2_-EIS structure with plasma treatment is similar to the response of the samples with chemical procedures, where the urea sensitivity are 105 ± 15 and 117 ± 9 mV/pUrea, respectively. The sensitivity value was the average value of five results. The C-V curves and the voltage shift in a linear range of these two methods are almost the same. The linearity of the calibration curves for both output voltages are very high and very suitable for physiological detection [15]. Based on these results, the chemical silanization method for urease immobilization is successfully replaced by remote NH_3 _plasma treatment, which has advantages of improving process safety, reducing environmental pollution, and lessening the process time. In addition, comparing the two methods, processing time can be reduced by almost 24 h by remote NH_3 _plasma treatment.

**Figure 4:**
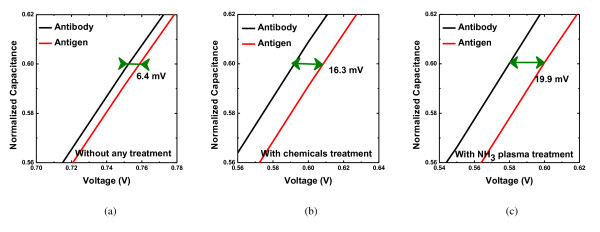
**The BSA detection response of ALD-HfO_2_-EIS structure**. (**a**) Without any treatment, (**b**) with chemical silanization treatment, and (**c**) with NH_3 _plasma treatment.

Moreover, the replacement of silanization procedure using NH_3 _plasma was also performed on the immobilization process of anti-BSA. Figure 4a shows that the response signal of the EIS membrane without any modification is 6.4 mV. The detection responses of chemical silanization and NH_3 _plasma treatment are 16.8 and 19.9 mV, respectively. The result indicates that the response of remote NH_3 _plasma is much higher than that of without plasma treatment. The results show that the NH_3 _plasma treatment is suitable and attractive for bio-sensing application.

## Conclusions

In this work, we investigated the effect of thickness on the pH sensitivity of thin HfO_2 _films formed by ALD based on the EIS structure. Moreover, a simple remote NH_3 _plasma treatment developed on an ALD-HfO_2 _membrane to replace the complicated silanization procedure for biomolecular immobilization in a covalent bonding method was proposed. Promising results in urea and antigen detections were obtained. They indicated that the remote NH_3 _plasma treatment is an attractive method to form the NH_2 _group on the membrane surface, suggesting an excellent potential on bio-sensing application.

## Competing interests

The authors declare that they have no competing interests.

## Authors' contributions

I-SW and T-FL executed the experiments, participated in the data analysis, and drafted and wrote the manuscript. C-EL and C-HH participated in the data analysis and optimized the structure of the manuscript. PY and Y-TL participated in the data analysis and executed the antibody immobilization. DGP provided the method of immobilization. C-MY participated in the data analysis. J-CW, J-SY, Y-SC, CC, and the corresponding author C-SL conceived and guided this study, integrated the analysis, and supplied the materials and instruments in this experiment. All authors read and approved the final manuscript.
